# Supplementary material for: Genome Assemblies for Seven Families of Birds From the Global South
Source: Mol Ecol Resour. 2026 Jul 15;26(5):e70162. doi: 10.1111/1755-0998.70162 (PMC13373495; doi:10.1111/1755-0998.70162)
Supplement: Supplementary file 5 — Figure S1: GenomeScope 2.0 profile of the k‐mer spectra at k = 2o for the seven species obtained with Jellyfish using short reads. k‐mer profile matches that of low (< 1%) heterozygosity. The bird silhouette is generated using a photo from Wikimedia Commons under a CC BY‐SA 4.0 licence. Figure S2: Coverage histogram generated by purge_haplotigs using the ‘hist’ function from the draught assembly and Oxford Nanopore raw reads. The bird silhouette is generated using a photo from Wikimedia Commons under a CC BY‐SA 4.0 licence. Figure S3: SnailPlots generated by BlobtoolKit indicate seven assemblies' genome characteristics. The circle plot represents the total size of the assembly. From the inside out, the central plot covers length‐related metrics. The red line represents the size of the longest scaffold; all other scaffolds are arranged in size order, moving clockwise around the plot. Dark and light orange arcs show the scaffold N50 and scaffold N90 values. The dark versus light blue area around it shows mean, maximum and minimum GC versus AT content. BUSCO scores are obtained from the Compleasm. The bird silhouette is generated using a photo from Wikimedia Commons under a CC BY‐SA 4.0 licence. Figure S4: Stacked bar plots of genome annotations of assemblies. Each bar represents a different category of annotations. Table S1: Reference seed used for the mitochondrial genome identified by MitoHiFi. Table S2: Characteristics of the de novo assembled and annotated mitogenomes. [file MEN-26-e70162-s002.docx]

**Supplemental Information for:**

Genome assemblies for seven families of birds from the Global South

Vinay K L^1,*^, Naman Goyal^2^, Ashwin Warudkar^2^, Chiti Arvind^2^, Robin V. V.^2,*^

^1^ Louisiana State University, Department of Biological Sciences, Baton Rouge, LA, USA.

^2^ Indian Institute of Science Education and Research Tirupati, Tirupati, Andhra Pradesh, India.

**Table of Contents:**

| **Figure S1** | Page 2 |
| --- | --- |
| **Figure S2** | Page 3 |
| **Figure S3** | Page 4 |
| **Figure S4** | Page 5 |
| **Table S1** | Page 6 |
| **Table S2** | Page 6 |

**
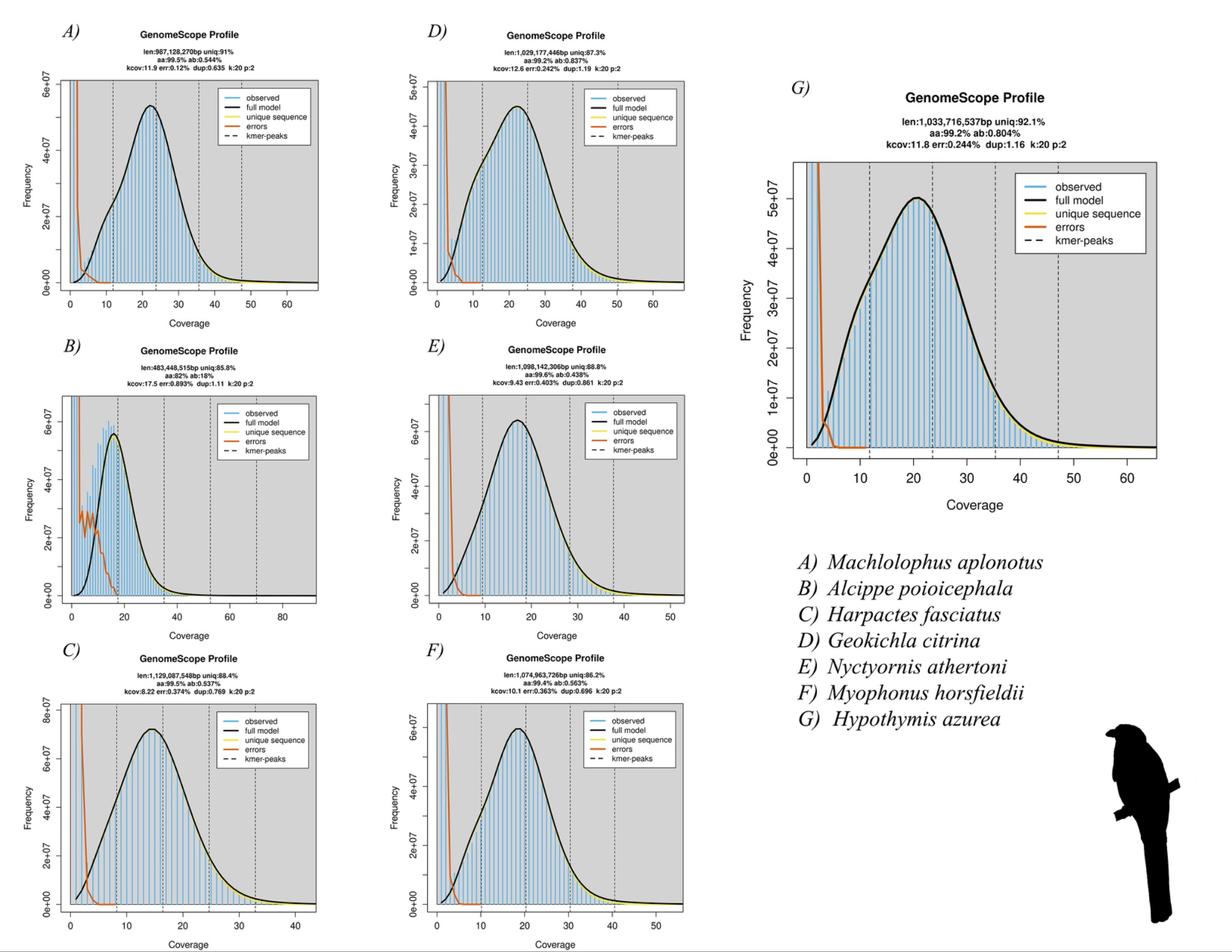
**

**Figure S1:** GenomeScope 2.0 profile of the k-mer spectra at k = 2o for the seven species obtained with Jellyfish using short reads. k-mer profile matches that of low (<1%) heterozygosity. The bird silhouette is generated using a photo from Wikimedia Commons under a CC BY-SA 4.0 license.

**
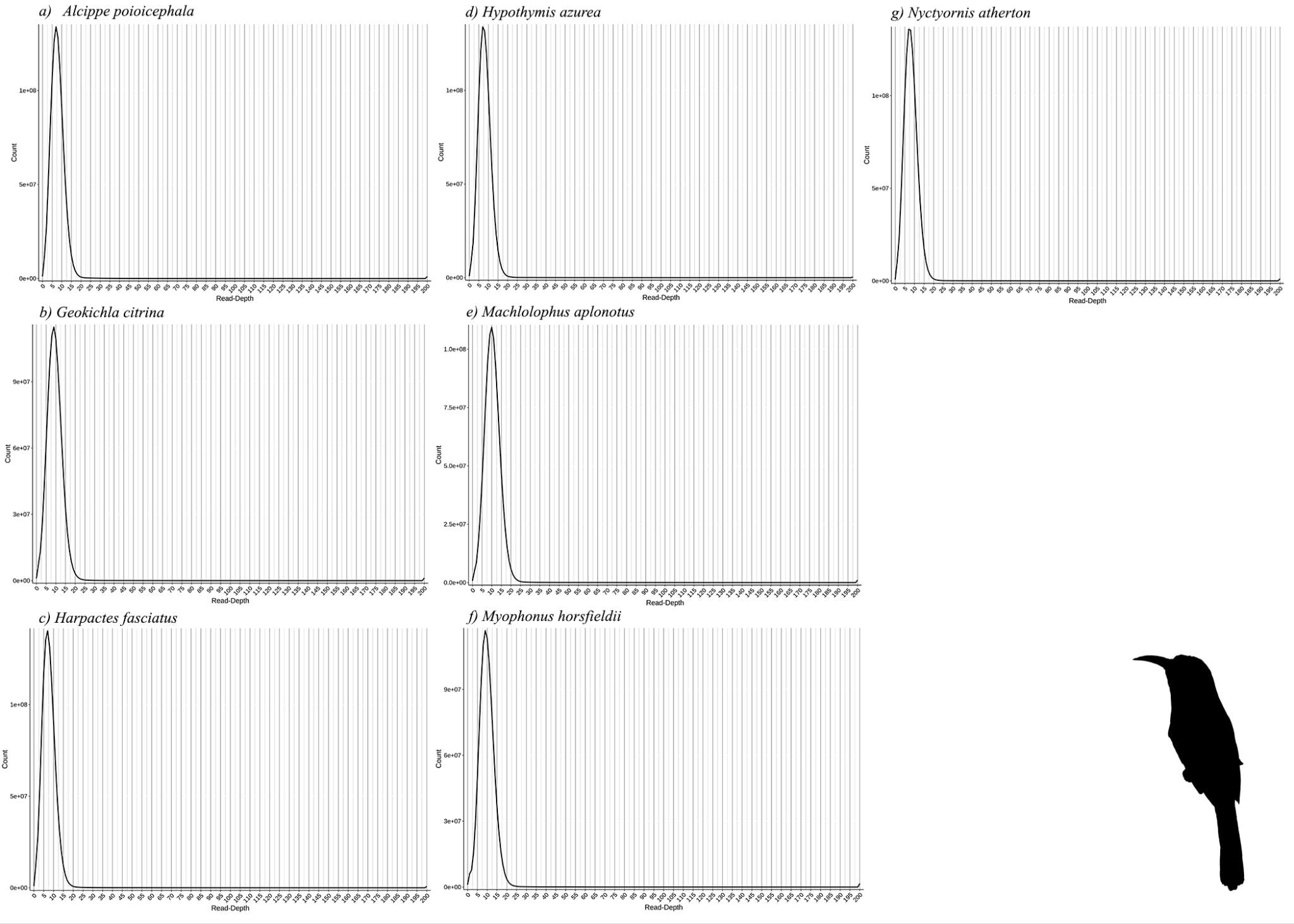
**

**Figure S2:** Coverage histogram generated by purge_haplotigs using the ‘hist’ function from the draft assembly and Oxford Nanopore raw reads. The bird silhouette is generated using a photo from Wikimedia Commons under a CC BY-SA 4.0 license.


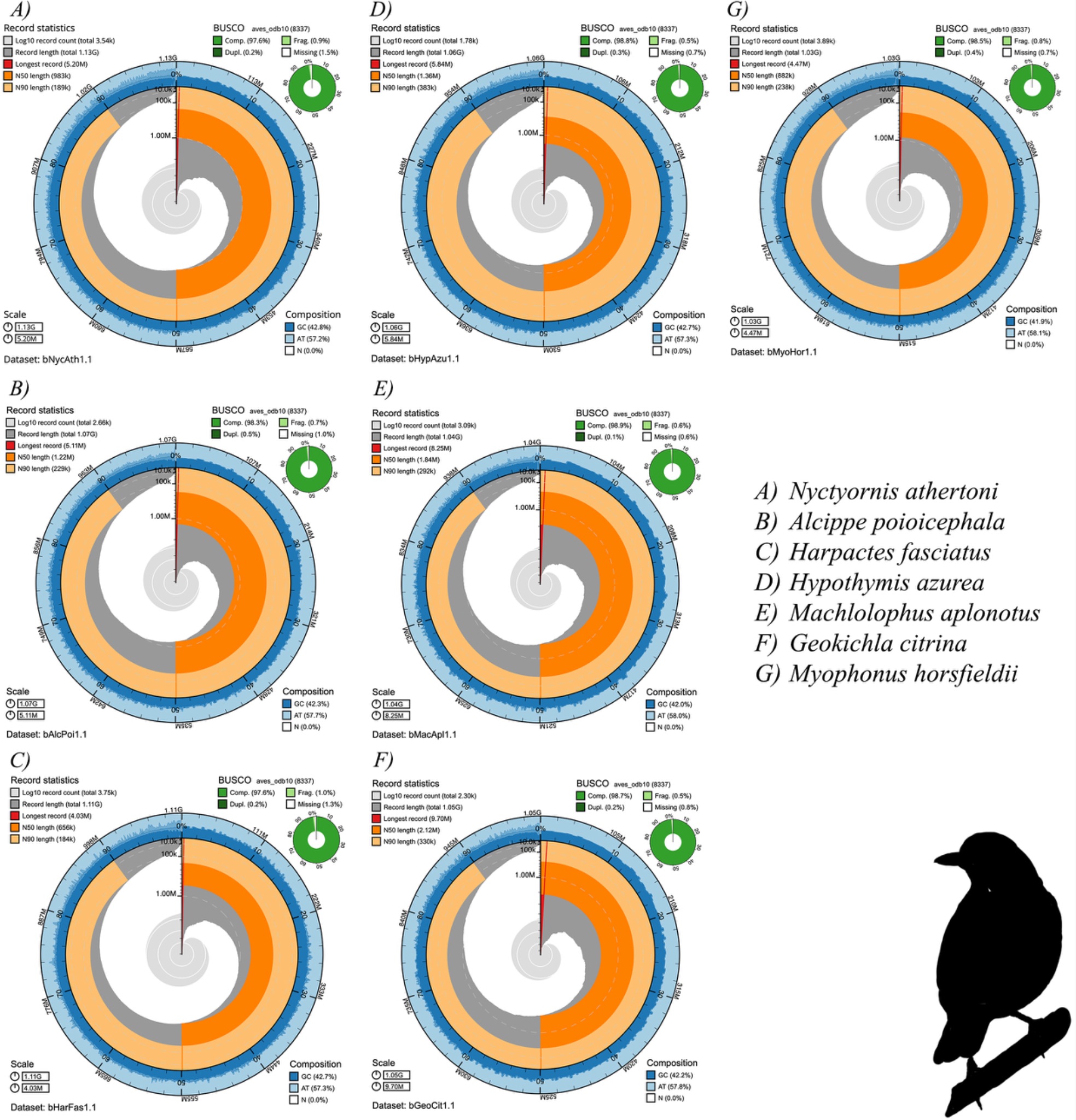


**Figure S3:** SnailPlots generated by BlobtoolKit indicate seven assemblies' genome characteristics. The circle plot represents the total size of the assembly. From the inside out, the central plot covers length-related metrics. The red line represents the size of the longest scaffold; all other scaffolds are arranged in size order, moving clockwise around the plot. Dark and light orange arcs show the scaffold N50 and scaffold N90 values. The dark versus light blue area around it shows mean, maximum, and minimum GC versus AT content. BUSCO scores are obtained from the Compleasm. The bird silhouette is generated using a photo from Wikimedia Commons under a CC BY-SA 4.0 license.


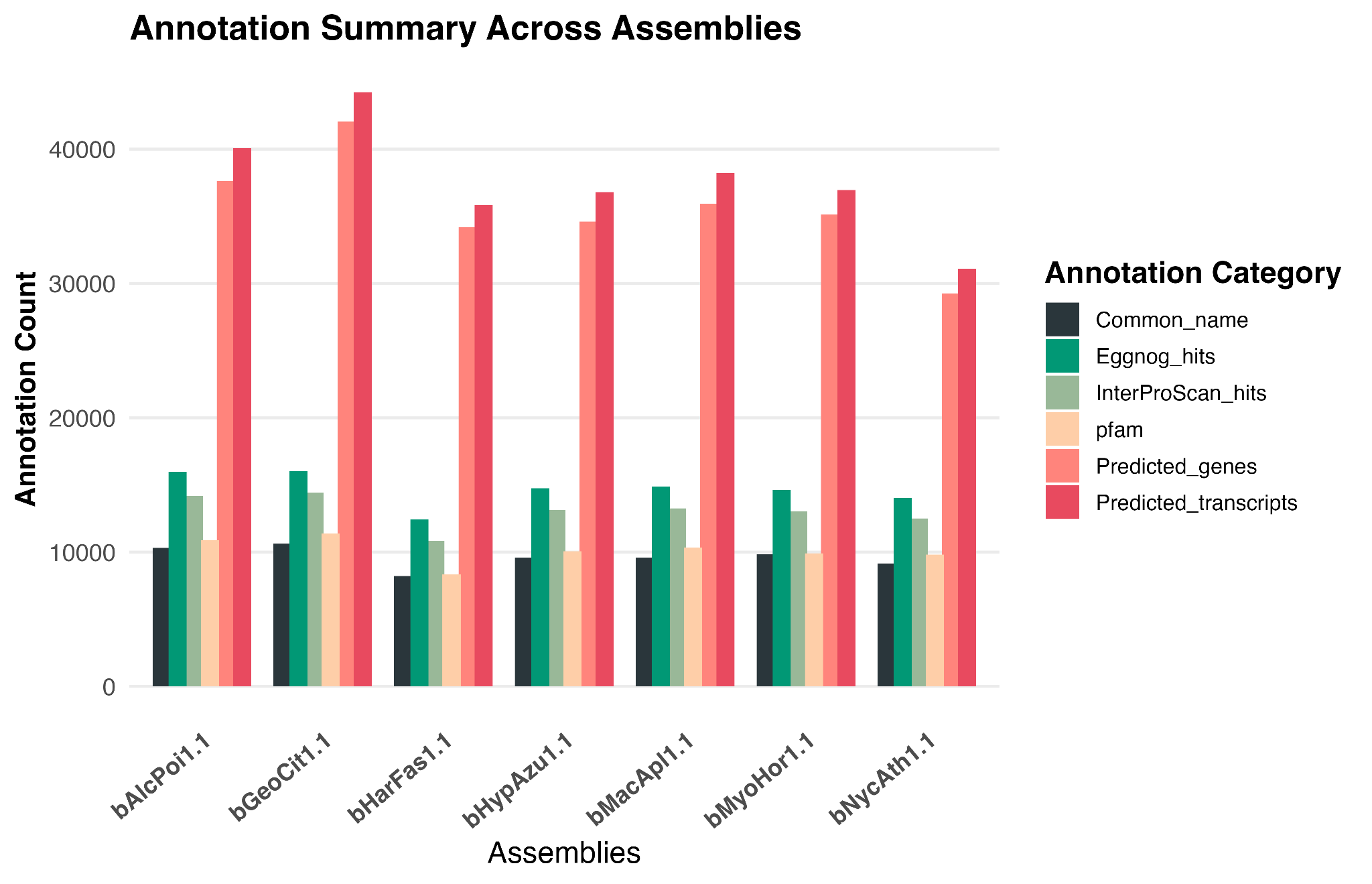


**Figure S4**: Stacked bar plots of genome annotations of assemblies. Each bar represents a different category of annotations.

**Table S1:** Reference seed used for the mitochondrial genome identified by MitoHiFi

| **Species** | **Seed Accession** | **Reference Species** |
| --- | --- | --- |
| *Alcippe poioicephala* | KX376475.1 | *Alcippe hueti* |
| *Geokichla citrina* | MK377247.1 | *Geokichla sibirica* |
| *Harpactes fasciatus* | NC_052802.1 | *Trogon melanurus* |
| *Hypothymis azurea* | NC_059916.1 | *Grallina cyanoleuca* |
| *Machlolophus aplonotus* | KX388476.1 | *Machlolophus spilonotus* |
| *Myophonus horsfieldii* | PP070394.1 | *Myophonus caeruleus* |
| *Nyctyornis athertoni* | NC_034642.1 | *Merops viridis* |

**Table S2:** Characteristics of the de novo assembled and annotated mitogenomes.

| **Species** | **Size (bp)** | **Protein coding** | **rRNAs** | **tRNAs** | **GC %** |
| --- | --- | --- | --- | --- | --- |
| *Nyctyornis athertoni* | 16,203 | 12 | 2 | 20 | 47 |
| *Hypothymis azurea* | 14,066 | 12 | 1 | 19 | 45 |
| *Machlolophus aplonotus* | 15,603 | 13 | 2 | 22 | 49 |
| *Myophonus horsfieldii* | 16,830 | 13 | 2 | 22 | 46 |
| *Harpactes fasciatus* | 15,474 | 12 | 2 | 20 | 43 |
| *Geokichla citrina* | 16,187 | 13 | 2 | 21 | 46.1 |
| *Alcippie poioicephla* | 17,888 | 13 | 2 | 22 | 46.1 |
